# Comparative Study of the Thermal and Hydraulic Performance of Supercritical CO_2_ and Water in Microchannels Based on Entropy Generation

**DOI:** 10.3390/e24091312

**Published:** 2022-09-16

**Authors:** Yi Tu, Yu Zeng

**Affiliations:** 1 Hunan Key Laboratory of Distributed Electric Propulsion Vehicle Control Technology, Hunan University of Arts and Sciences, Changde 415000, China; 2 School of Aeronautic Science and Engineering, Beihang University, Beijing 100191, China

**Keywords:** heat transfer, entropy generation rate, supercritical CO_2_, computational fluid dynamics, microchannel

## Abstract

The excellent thermophysical properties of supercritical CO_2_ (sCO_2_) close to the pseudocritical point make it possible to replace water as the coolant of microchannels in application of a high heat flux radiator. The computational fluid dynamics (CFD) method verified by experimental data is used to make a comparison of the thermal hydraulic behavior in CO_2_-cooled and of water-cooled microchannels. The operation conditions of the CO_2_-based cooling cases cover the pseudocritical point (with the inlet temperature range of 306~320 K and the working pressure of 8 MPa), and the water-based cooling case has an inlet temperature of 308 K at the working pressure of 0.1 MPa. The channel types include the straight and zigzag microchannels with 90°, 120°, and 150° bending angles, respectively. The analysis result shows that, only when the state of CO_2_ is close to the pseudocritical point, the sCO_2_-cooled microchannel is of a higher average heat convection coefficient and a lower average temperature of the heated surface compared to the water-cooled microchannel. The entropy generation rate of the sCO_2_-cooled microchannel can reach 0.58~0.69 times that of the entropy generation rate for the water-cooled microchannel. Adopting the zigzag structure can enhance the heat transfer, but it does not improve the comprehensive performance represented by the entropy generation rate in the sCO_2_-cooled microchannel.

## 1. Introduction

With the development of electronic equipment in miniaturization and high integration, high heat flux generation poses a greater challenge to the heat dissipation of equipment. There are various potential solutions that have been proposed as potential candidates for electronic cooling, such as impinging jet cooling technology [1,2], heat pipes [3,4], the application of porous materials which can provide a high heat exchange area to volume ratio [5,6,7], as well as microchannel heat exchangers. The use of microchannels is one of the most important solutions for the design of compact heat sinks for high heat flux removal. Tuckerman and Pease [8] first proposed and tested microchannel heat exchangers and realized heat dissipation with 790 W/cm^2^ for a silicon integrated circuit using water as a coolant. Although microchannel heat exchangers have a surprising heat dissipation capacity, they also have high pumping power requirements due to the high flow resistance of the microchannels [9].

Investigations have been conducted to improve the thermo-hydraulic performance of microchannel heat exchangers, focusing mainly on two directions: one to improve the geometric structure of the microchannel, and the other to find high-performance coolants. Mohammed Adham [10] and Khoshvaght-Aliabadi [11] have investigated the effect of the geometry of the microchannel heat exchanger on its hydraulic and heat dissipation performance using water as coolant. In their research, the types of triangular, trapezoidal, and sinusoidal channels are compared, and the results indicated that the sinusoidal channel is of the top value of the ratio of average heat convection coefficient to power of pump. Xu et al. [12] suggested a kind of multilayer fractal silicon-based microchannel to optimize the overall pressure drop of the microchannel through step-by-step bifurcation and stratification. Wang et al. [13] studied the effect of geometry parameters on the thermo-hydraulic performance of rectangular microchannels, such as aspect ratio, and found that microchannels show optimum effects with an aspect ratio between 8.904 and 11.442. Song et al. [14] also conducted similar research on trapezoidal microchannels. Kim [15] investigated the validity of the *Nu* correlation based on the normal channel size to analyze the thermo-hydraulic performance of the microchannel using water as a coolant. The research result showed that the predicted value of *Nu* by the traditional theoretical correlation model is only reasonable if *Re* > 180 and the aspect ratio >1. Peng et al. [16] examined the thermal and hydraulic performance of the zigzag experiment microchannels at four different angles (30°, 45°, 60°, 90°) using deionized water as the working fluid. Numerical analysis with experimental verification is also an important and efficient research method. B. Xu et al. [17] tested the flow characteristics in microchannels with hydraulic diameters ranging from 30 μm to 344 μm, and the analysis results showed that the Navier–Stokes formula is able to precisely predict the flow characteristics in the microchannel. Ramos-Alvarado et al. [18] deeply analyzed the impact of channel configurations on the pressure loss as well as the uniformity of the microchannel heat exchanger temperature based on the CFD method. Sohankar et al. [19] investigated how the aspect ratio of the cross section numerically affects the thermal hydraulic characteristics of the rotating rectangular U-shaped microchannel.

To improve the thermo-hydraulic performance of microchannel radiators, the use of better performance coolants is also one of the key research directions. The application of nanofluid as a cooling medium is one of the major branches of research. In this line of study, the heat transfer characteristics of various nanofluids in microchannels have been studied, including Al_2_O_3_ [20], CuO [21], TiO_2_ [22], Cu-Al_2_O_3_ [23], and ZnO nanofluid [24]. Chein and Chuang [25] studied the performance of a microchannel radiator with a CuO-H_2_O mixed coolant. Their research indicates that nanofluids can absorb more heat than water cooling when both are at low flow rates, while in the case of high flow, the thermal performance is mainly determined by the volumetric flow rate, in addition to that the nanoparticles have little effect in this process. Jung and Park [26] conducted an experimental comparative investigation on the thermo-hydraulic performance of Al_2_O_3_-water nanofluid in microchannels. Their results indicated that the entropy generation rate of nanofluid is 6.3% lower than that of water, while water has a lower flow resistance. The result also indicated that nanofluids present preferable heat transfer characteristics to water, but their channel pressure loss is not dominant compared to that of water.

**Figure 1:**
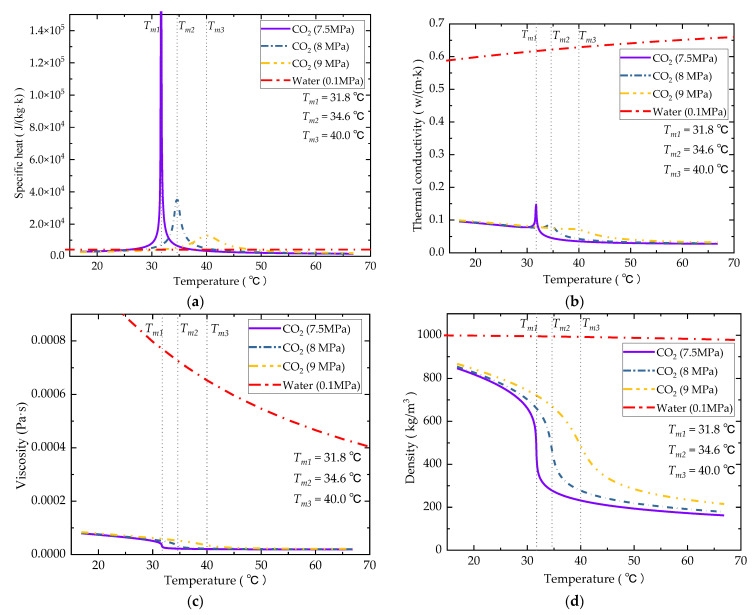
Thermophysical properties of CO_2_ at different working pressure (7.5, 8.0, and 9 MPa). (**a**) Specific heat; (**b**) thermal conductivity; (**c**) dynamic viscosity; (**d**) density.

CO_2_ is a coolant with excellent potential for application in high heat flux dissipation. As a coolant, CO_2_ possesses high thermal conductivity and specific thermal conductivity near the pseudocritical point, which is conducive for improving the heat transfer performance [27,28]. Furthermore, as shown in Figure 1, where the data are derived from the RERPROP V9.1 database, the density of CO_2_ near the pseudocritical point stays close to that of water, but its dynamic viscosity is significantly lower. These thermophysical characteristics of CO_2_ show the potential to improve flow resistance characteristics while maintaining a heat transfer performance close to that of water. Research in [29,30] both showed that supercritical CO_2_ (sCO_2_) can provide a higher cooling performance with lower channel pressure loss than liquid cooling. Khalesi and Sarunac [31] conducted a good deal of analyses on the development process and conjugated heat transfer in microchannels using supercritical CO_2_ and liquid sodium as coolants. Their results showed that the sharp change in the thermophysical property of sCO_2_ near the pseudocritical point will impact the flow and thermo-hydraulic performance in the microchannels, and this effect will be weakened with the operational conditions being further away from the critical point. 

The results of existing research show that the sCO_2_ coolant can provide a higher cooling performance with a lower channel pressure loss compared to the liquid coolant. However, since the thermophysical properties of CO_2_ vary sharply near the pseudocritical point, the impact of fluid temperature and pressure change in the microchannel on its thermo-hydraulic performance has not been clearly clarified in existing research. The density of sCO_2_ is lower than water, and the pump power consumption of the microchannel heat exchanger is also affected by the density of the fluid, which is one of the parameters that affects the comprehensive performance of the microchannel heat exchanger. In high heat flux heat dissipation applications, it is often desirable to achieve better heat transfer performance with smaller package volume and lower flow resistance (pump power consumption). In this paper, the flow and heat transfer performance of water and CO_2_ coolants in microchannels are comprehensively compared based on the entropy generation rate. Special channels such as zigzag [32], curve [33], and trapezoid [34] are important ways to improve thermal performance in the microchannel. A comparison analysis of the effect of the bend angle on the thermal hydraulic performance is also conducted in this study for water and sCO_2_-cooled channels.

## 2. Numerical Method

In this study, water and sCO_2_ are used as coolants to study their thermo-hydraulic performance in microchannels. The numerical simulation employs the software ANSYS fluent V19.0 to solve the conjugate heat transfer problem and the Navier–Stokes equations for the computational domains. The SST-kω model was used to model turbulence. The pressure-based solver with pressure–velocity coupling was used to solve the flow problem through the ANSYS FLUENT package. The thermophysical characteristics of CO_2_ are based on the NIST real gas model with the REFPROP V9.1 database.

### 2.1. Physical Model and Boundary Conditions

**Figure 2:**
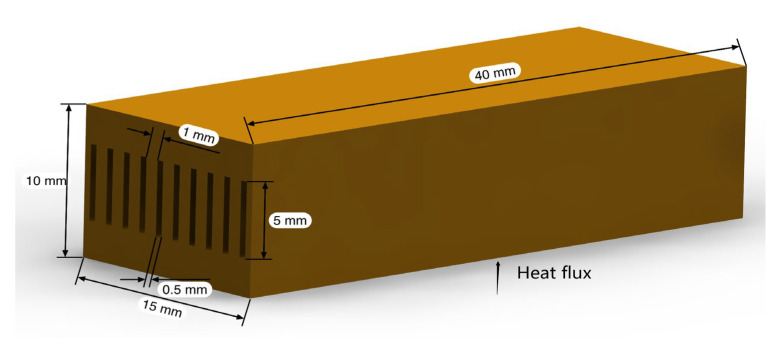
Physical model.

The geometrical model of the microchannel radiator in this analysis is illustrated in Figure 2. The material of this radiator is copper and there are 10 microchannels in total. The width, height, and depth of the radiator is 15 mm × 10 mm × 40 mm. The width and height of the fluid domain is 0.5 mm × 5 mm. In this study, it is assumed that the design of the heat sink header can ensure sufficient uniformity of the fluid in each channel.

The working boundary conditions for the two fluids are given in Table 1. To make CO_2_ work near the pseudocritical point, the outlet pressure of the channels is set at 8 MPa. In this study, a variety of input temperatures (306 K to 320 K) are used to understand the impact of deviation from the pseudocritical point on its thermo-hydraulic performance. Since the temperature and pressure of liquid water have a minor effect on its thermophysical properties, a single inlet temperature (308 K) and a single outlet pressure (0.1 MPa) are adopted. The uniform flow inlet boundary with variation range of mass flux 50~1000 kg/(m^2^·s) are adopted for both fluid-type conditions. The heat flux ranges from 40,000 to 120,000 W/m^2^ for the heated surface on the bottom side of the solid domain, the coupled heat transfer boundary is adopted at the interface between the fluid and solid regions, and the adiabatic boundary is used for the rest surfaces of the solid domain.

### 2.2. Governing Equations and Data Reduction

The continuity, momentum, and energy equations for steady-state flow of the computation fluid domain are expressed as Equations (1)–(3) [35], which are solved by the commercial code ANSYS Fluent. Radiation is not considered in the energy solution. The buoyancy effect is ignored in this analysis of internal forced convection, as the Richardson number is far less than 0.1 even under the condition of minimum mass flux.

(1) $$\frac{\partial }{\partial x_{i}}\rho u_{i}=0$$

(2) $$\frac{\partial }{\partial x_{j}}\rho u_{i}u_{j}=-\frac{\partial p}{\partial x_{i}}+\frac{\partial \tau_{ij}}{\partial x_{j}}$$

(3) $$\frac{\partial }{\partial x_{i}}\left(u_{i}\left(\rho E+p\right)\right)=\frac{\partial }{\partial x_{i}}\left(k_{eff}\frac{\partial T}{\partial x_{i}}\right)$$

where $u_{i}$ is overall velocity vector, *E* is the total energy, $\tau_{ij}$ is the stress tensor, and $k_{eff}$ is the effective conductivity ($k_{eff}=k+k_{t}$, $k_{t}$ is the turbulent thermal conductivity).

In the solid region the energy transport equation for steady state is expressed as Equation (4):

(4) $$\frac{\partial }{\partial x_{i}}\left(k_{sl}\frac{\partial T}{\partial x_{i}}\right)=0$$

where $k_{sl}$ is the thermal conductivity of the solid material.

To evaluate the heat transfer performance of microchannel heat sink, the average heat convection coefficient, *h*, is given in Equation (5), where $Q_{w}$ uses the total heat transfer rate of the wall surface.

(5) $$h=\frac{Q_{w}}{T_{w}-T_{b}}$$

The Nusselt number is defined by Equation (6):

(6) $$Nu=\frac{hd_{h}}{k}$$

Hydraulic diameter is calculated through Equation (7):

(7) $$d_{h}=\frac{4A}{C}$$

The total pressure drop of the fluid in the microchannel is defined as Equation (8):

(8) $$\Delta P=P_{in}-P_{out}$$

where $P_{in}$ and $P_{out}$ use the area weighted average value obtained from the CFD results at the entrance and exit plane of the microchannel.

The pump power to drive fluid flow in microchannels, *W*, is calculated by Equation (9):

(9) $$W=\frac{\dot{m}}{\rho }\Delta P$$

There are irreversible losses in the flow and heat transfer process of the coolant in the microchannel. The irreversible losses contain two parts: One part is the irreversible loss caused by heat transfer driven by the temperature difference, which is expressed by ${\dot{S}}_{g,\Delta T}$ as shown in Equation (10). Under the same heat flux, the smaller the temperature difference during the heat transfer process, the higher the average heat convection coefficient of the microchannel, and the corresponding ${\dot{S}}_{g,\Delta T}$ is also lower. The second part is the irreversible loss caused by the frictional flow in the microchannel, which is expressed by ${\dot{S}}_{g,\Delta P}$ as shown in Equation (11), and this loss is directly related to the pumping power required to drive the same mass flow fluid. In this paper, the total entropy generation rate ${\dot{S}}_{g}$ in the heat transfer process as shown in Equation (12) is used to evaluate the comprehensive performance of the microchannel by assuming that the fluid is in a stable flow state and the temperature change along the length of the channel is much smaller than the core temperature of the fluid [36,37].

(10) $${\dot{S}}_{g,\Delta T}=\frac{q_{w}A\left(T_{w}-T_{b}\right)}{T_{w}T_{b}}$$

(11) $${\dot{S}}_{g,\Delta P}=\frac{\dot{m}}{\rho_{b}T_{b}}\Delta P$$

(12) $${\dot{S}}_{g}=\frac{q_{w}A\left(T_{w}-T_{b}\right)}{T_{w}T_{b}}+\frac{\dot{m}}{\rho_{b}T_{b}}\Delta P$$

### 2.3. Mesh Description and Independence

**Figure 3:**
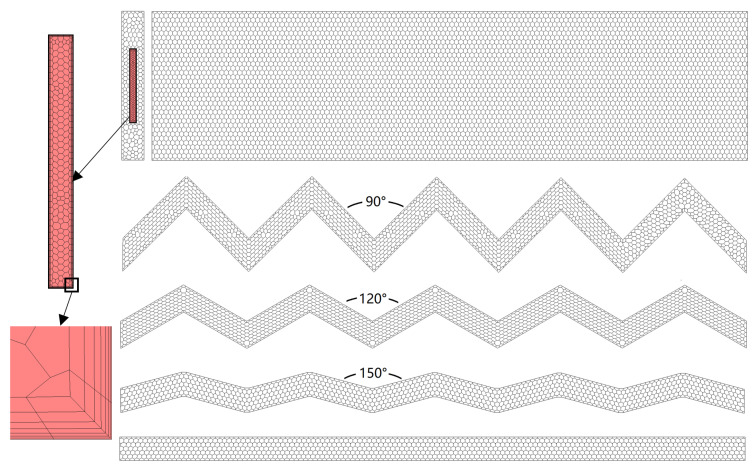
Mesh models.

The physical model consists of 10 flow channels. According to the principle of symmetry, one of them is intercepted for analysis. In this analysis, STAR-CCM+ was used to build polyhedral meshes with cylindrical prism layers. As shown in Figure 3, the mesh of the fluid region was finely constructed with 7 wall prism layers, making the wall Y+ < 1, and the relatively coarse mesh is adopted for the solid region. The mesh sizes of 287,842, 552,704, and 764,002 were applied to conduct the mesh independence analysis. 

**Figure 4:**
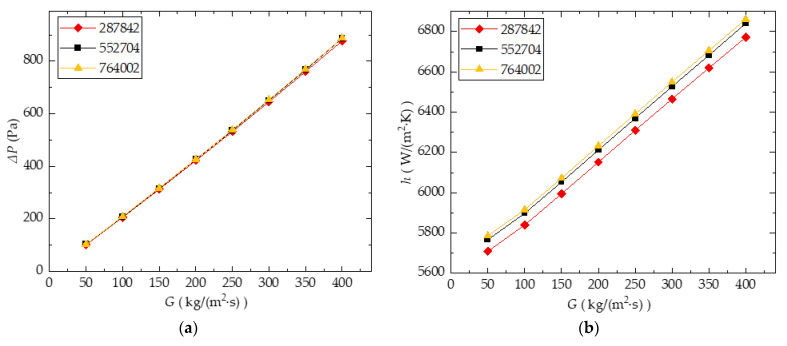
Mesh independent analysis result: (**a**) ΔP; (**b**) h.

Figure 4a,b show the comparison of ΔP and h curves for all three mesh size cases. As illustrated in this result, the error in the ΔP and h between the mesh sizes 552,704 and 764,002 is quite small. Taking into account the computational accuracy and efficiency, the rest of the study used 500,000 meshes as the baseline. 

### 2.4. Validation 

**Figure 5:**
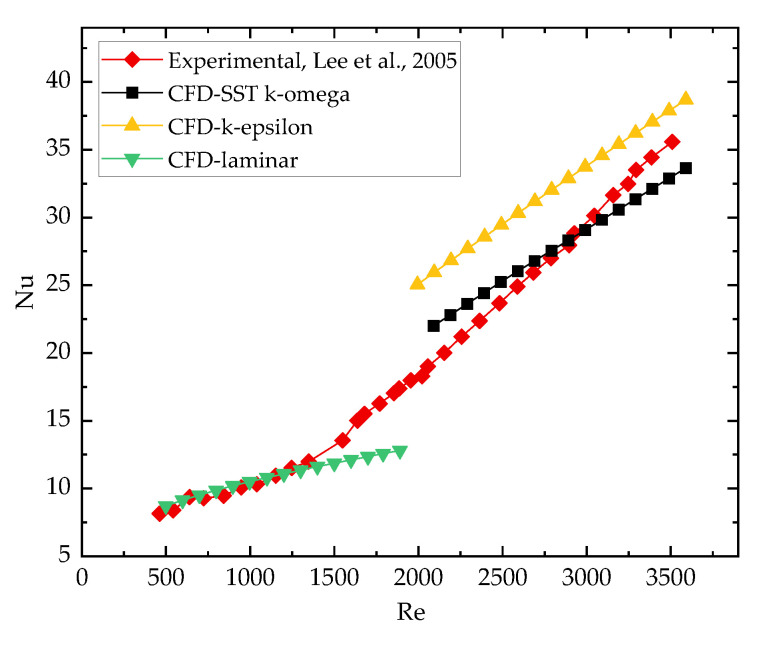
Validation with experimental data [38].

To further validate the feasibility of the numerical method, in this study, the numerical results with the SST k-omega and k-epsilon turbulent models are compared with the test data obtained in [38]. The comparison was conducted under water-cooled condition with an *Re* range of 500~3500 (sCO_2_ as a coolant is detailed in [39]). The comparison result in Figure 5 shows that the numerical result maintained consistency with the experiment data well in the linear flow state when *Re* < 1500. When *Re* > 2000, compared with the k-epsilon turbulence model (with maximum relative error 33.8%), the calculation result of the k-omega turbulent model of shear stress transfer (with maximum relative error 13.7%) shows a better consistency with the experiment data. In the transitional region from laminar to turbulent flow, 1500 < *Re* < 2000, the maximum relative error is 26.5% using laminar models. Since the maximum Reynolds numbers of analysis cases in the following study can reach more than 5000, the k-omega SST model will be adopted for turbulent flow cases.

## 3. Results and Discussions

By comparing the thermophysical properties of water and sCO_2_, it is apparent that the thermal conductivity of CO_2_ is lower than that of water by no more than one order of magnitude. Nevertheless, the specific heat capacity of sCO_2_ shows several orders of magnitude higher than that of water, which is beneficial for its heat transfer performance in microchannels. The dynamic viscosity of sCO_2_ is also lower than that of water by several orders of magnitude, which is significantly beneficial for its flow characteristics in microchannels. In this analysis, the thermal and hydraulic characteristics of water and sCO_2_ in straight and zigzag microchannels are compared and analyzed to understand the feasibility of replacing water with sCO_2_ as a coolant.

### 3.1. Comparative Study of Straight Channel Cases in Different Mass Flux

In this section, a comparative study is conducted on the following four parameters, ΔP, *h*, *T_hsur_*, and ${\dot{S}}_{g}$, to analyze the thermal and hydraulic performance of water and sCO_2_ in microchannels. Six inlet temperatures of 306 K, 307 K, 308 K, 310 K, 315 K, and 320 K are used for the analysis of CO_2_. For liquid water, the inlet temperature is 308 K. The inlet mass flux range of the two coolants is 50~1000 kg/(m^2^·s). All cases adopt the pressure outlet boundary. The outlet pressure is 8 MPa for CO_2_-cooled cases and 0.1 MPa for water-cooled cases. The constant heat flux between 4 and 120 W/cm was adopted for the heat surface of the microchannel.

#### 3.1.1. Pressure Drop

**Figure 6:**
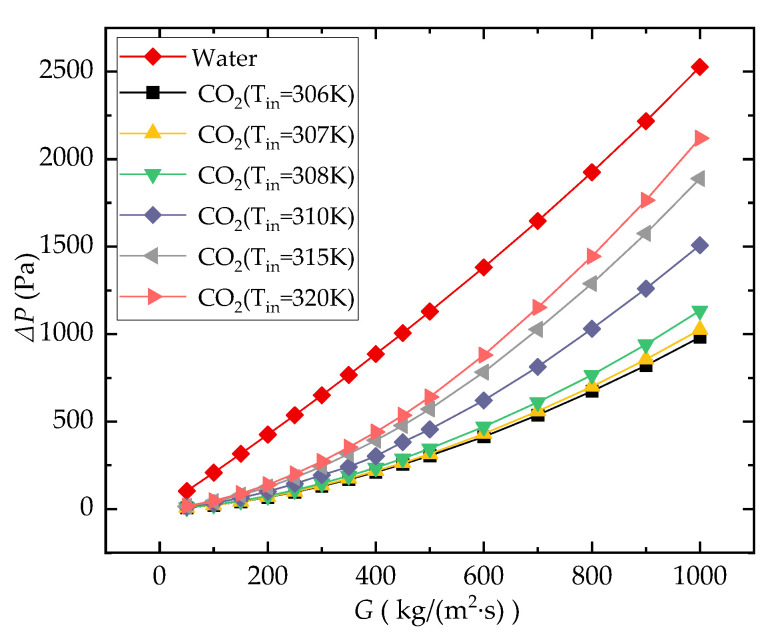
Comparison of Δ*P* of the microchannel (*q_w_* = 40,000 W/m^2^).

It is obvious as illustrated in Figure 6 that the Δ*P* curve of the water-cooled microchannel is significantly higher than that of the CO_2_-cooled microchannel at each inlet temperature. Obviously, this is in line with our predictions. This is because the dynamic viscosity of water at room temperature is significantly higher than that of sCO_2_ with its condition close to the pseudocritical state. For sCO_2_-cooled cases in this comparison analysis, the channel Δ*P* increases with increasing *T_in_*. This is because the Δ*P* through the microchannel is influenced by the dynamic viscosity and density of the coolant. These two thermophysical parameters both decrease with increasing *T_b_*, but the influence of density change takes the lead.

The pressure drop of the water-cooled channel is almost linear with the change in *G*, while this does not happen for the CO_2_-cooled one. This is also because the density and dynamic viscosity of CO_2_ change drastically with temperature near the pseudocritical point, especially density, which is much more sensitive to the temperature compared to the water. Similar conclusions also appeared in [30].

#### 3.1.2. Average Heat Convection Coefficient

**Figure 7:**
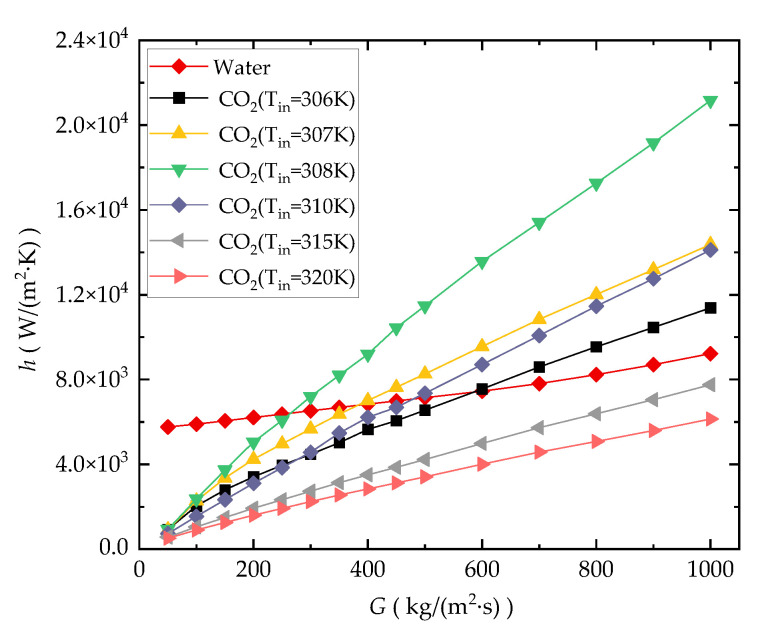
Comparison of *h* of the microchannel (*q_w_* = 40,000 W/m^2^).

**Figure 8:**
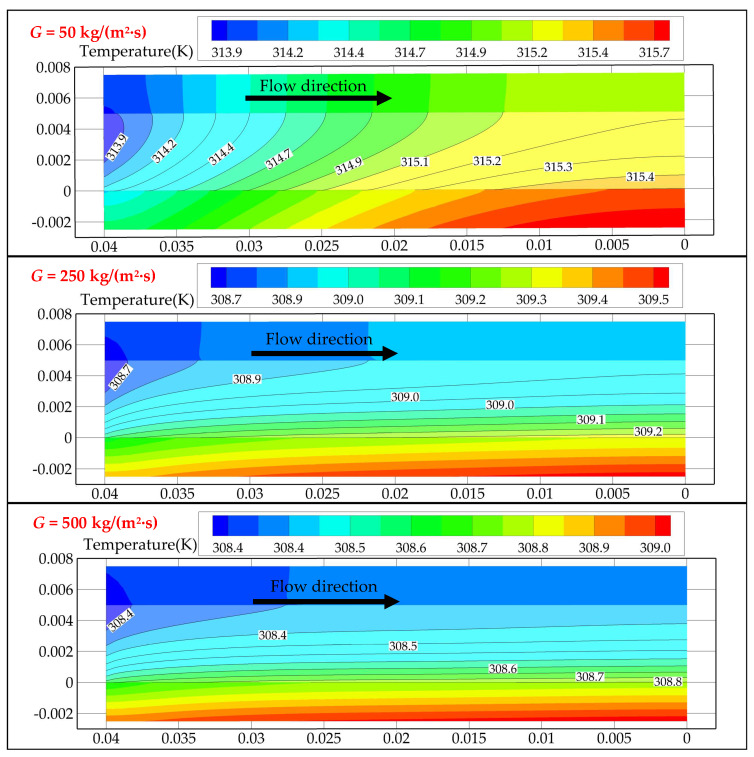
Changes in channel temperature along the flow direction.

As illustrated in Figure 7, *h* of the water-cooled cases is higher than those of sCO_2_-cooled cases with low mass flux (<250 kg/(m^2^·s)). This is because the bulk temperature *T_b_* of the fluid increases significantly relative to *T_in_* at low mass flow, resulting in a large deviation in *T_b_* from the pseudocritical temperature (*T_m_* = 307.75 K at 8 MPa) for CO_2_. This will lead to a sharp deterioration in the thermophysical (thermal conductivity and specific heat) properties of CO_2_. As the contour diagram in Figure 8 shows, the temperature change in the fluid along the channel decreases with the increase in *G*. Figure 7 also shows that *h* of water and CO_2_ both gradually increase with the increasing *G*, but the growth rate of the CO_2_-cooled case is larger. As *G* increases, the temperature variation of the CO_2_ fluid along the flow path becomes smaller, making its deviation from the pseudocritical point smaller. In this high mass flux condition, the *h* for water-cooled channels is not as good as *h* for the CO_2_-cooled channels in the cases where the *T_in_* is close to *T_m_* (*T_in_* = 306 K, 307 K, 308 K, 310 K). For CO_2_-cooled cases, the highest value, and the highest rate of increase in *h*, occur at *T_in_* = 308 K, which is closest to *T_m_*. This is because the closer the state of CO_2_ is to the pseudocritical point, the better its thermophysical properties, and the more intense the change in its thermophysical properties.

#### 3.1.3. Average Temperature of the Heated Wall

**Figure 9:**
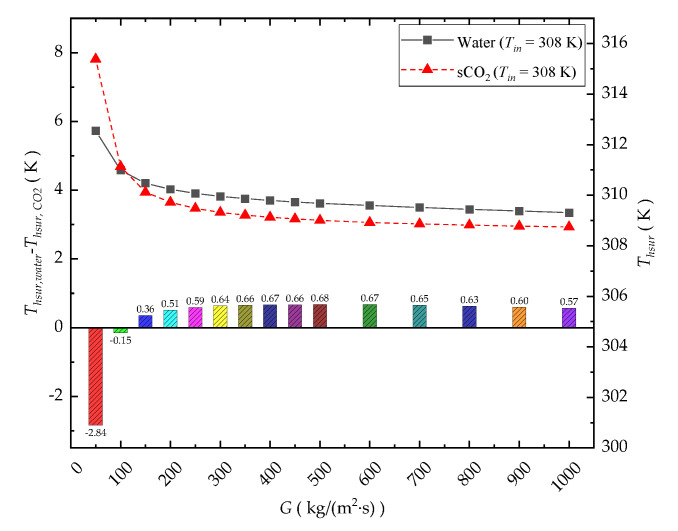
Comparison of *T_hsur_* of the microchannel.

Figure 9 shows the comparison result of the average temperature of the heated wall *T_hsur_* of the CO_2_-cooled and water-cooled microchannels. In this analysis, *T_in_* = 308 K, *q_w_* = 40,000 W/m^2^, *P_out_* = 8 MPa for the sCO_2_ case, *P_out_* = 0.1 MPa for the water case, and *G* = 50~500 kg/(m^2^·s). It also shows that the *T_hsur_* of the water-cooled channel is lower than that of the CO_2_-cooled channel only at an extremely low mass flux. This is also caused by the large deviation in the temperature of the CO_2_ fluid from *T_m_* under low mass flux conditions, resulting in the deterioration of its thermophysical properties and the weakening of the heat transfer performance. This impact will gradually decrease with increasing mass flux. When *G* > 100 kg/(m^2^·s), *T_hsur_* of the CO_2_-cooled channel will be lower than that of the water-cooled channel. When *G* > 300 kg/(m^2^·s), the average temperature difference in the heated surface between the two coolant-based cooling cases tends to be stable, and values of *T_hsur_* of the CO_2_-cooled channel are 0.5~0.7 K lower than those of the water-cooled channel.

#### 3.1.4. Entropy Generation Rate

**Figure 10:**
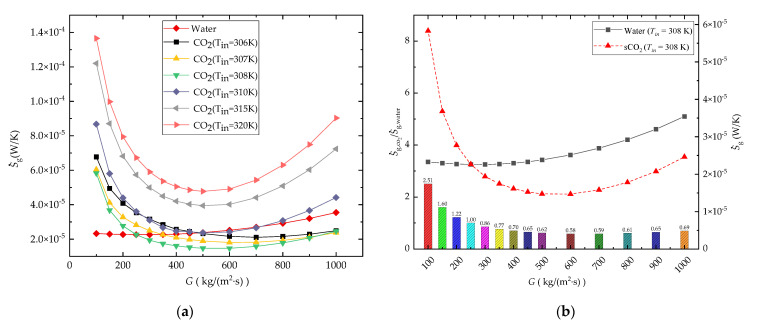
Comparison results of the entropy generation rate in different *G*: (**a**) ${\dot{S}}_{g}$; (**b**) $S_{g,CO_{2}}/S_{g,water}$.

Figure 10a,b shows the comparison of entropy generation rate ${\dot{S}}_{g}$ under different boundary conditions with water and CO_2_ as coolants at mass flux between 50~1000 kg/(m^2^·s) against different inlet temperatures (CO_2_: 306 K, 307 K, 308 K, 310 K, 315 K, 320 K; water: 308 K).

As shown in Figure 10a, the ${\dot{S}}_{g}$ of the CO_2_-cooled microchannel is lower than that of the water-cooled microchannel at relatively large mass flux in the case of *T_in_* = 306 K, 307 K, and 308 K, and the lowest ${\dot{S}}_{g}$ curve occurs when *T_in_* = 308 K. For the cases with *T_in_* = 315 K and 320 K, the ${\dot{S}}_{g}$ of the CO_2_-cooled microchannel is higher than that of the water-cooled microchannel due to the large deviation from the pseudocritical point. It also means that the comprehensive performance of the microchannels can be improved on the basis of maintaining the state of CO_2_ close to the pseudocritical point.

The ratio value of $S_{g,CO_{2}}$ at *T_in_* = 308 K to ${\dot{S}}_{g,water}$ in Figure 10b also shows that under the current working condition, when *G* < 250 kg/(m^2^·s) the performance of the CO_2_-cooled channel is not as good as that of the water-cooled channel, and when *G* > 500 kg/(m^2^·s) using sCO_2_ as a coolant can decrease the ${\dot{S}}_{g}$ to 0.58~0.69 times of the water-cooled straight microchannel.

**Figure 11:**
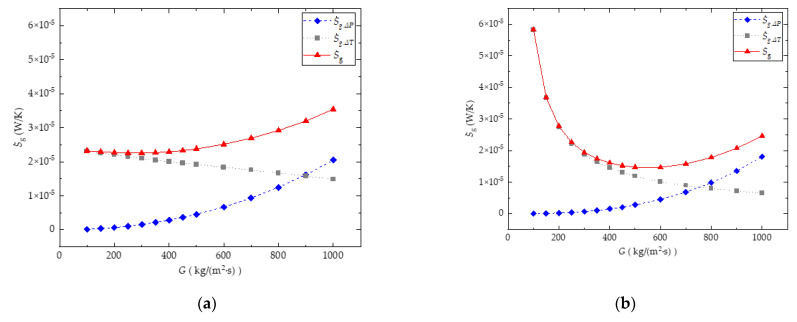
Effect of *G* on ${\dot{S}}_{g,\Delta P}$, ${\dot{S}}_{g,\Delta T}$, and ${\dot{S}}_{g}$: (**a**) water-cooled channel (*T_in_* = 308 K, *P_out_* = 0.1 MPa); (**b**) CO_2_-cooled channel (*T_in_* = 308 K, *P_out_* = 8 MPa).

It can also be seen from Figure 10a that there are minimum-value entropy generation rates for all water-cooled and sCO_2_-cooled cases within the current analyzed mass flux range. This is because as the mass flux increases, as shown in Figure 11a,b, for both the water-cooled and CO_2_-cooled channels, the ${\dot{S}}_{g,\Delta T}$ shows a decreasing trend due to the increasing heat convection coefficient in the microchannel, and ${\dot{S}}_{g,\Delta P}$ shows a increasing trend due to the increasing channel pressure drop. The total entropy generation rates ${\dot{S}}_{g}$ for both water-cooled and CO_2_-cooled cases decrease first, then increase continuously with the increasing *G*.

### 3.2. Comparative Study of Straight-Channel Cases in Different Heat Flux

It can be seen from the analysis in Section 3.1 that in order to ensure excellent heat transfer characteristics of the CO_2_ coolant, it is essential to maintain the CO_2_ state close to the pseudocritical point. With the increase in *q_w_*, the temperature change in the fluid along the channel will increase, causing the fluid state to deviate more from the pseudocritical point. This section studies the effect of heat flux (*q_w_*) on the h, ΔP, and ${\dot{S}}_{g}$ of straight microchannels cooled by water and CO_2_ for three different mass fluxes (500, 1000, 1500 kg/(m^2^·s)) at *T_in_* = 308 K, *P_out_* = 8 MPa for the sCO2 case, and *P_out_* = 0.1 MPa for the water case.

#### 3.2.1. Average Heat Convection Coefficient

**Figure 12:**
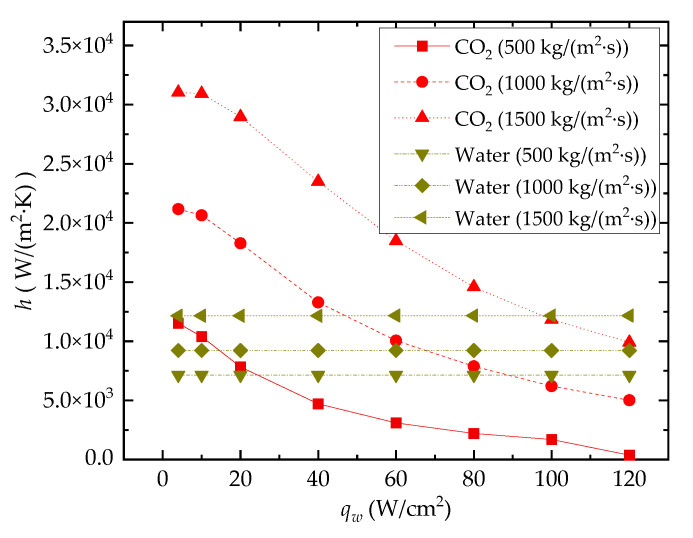
Effect of *q_w_* on *h* (*T_in_* = 308 K).

Figure 12 shows the effect of *q_w_* on heat transfer performance for both water-cooled and CO_2_-cooled channels. It can be seen from the result that the change in *q_w_* has little impact on *h* for water-cooled cases. This is because the change in coolant *T_b_* caused by the increase in *q_w_* has little effect on the thermophysical properties (specific heat and thermal conductivity) of the water. For the sCO_2_ coolant, however, as *T_in_* = 308 K, the fluid state is near the peak position of the curves of thermal conductivity and specific heat capacity. At this position, the values of both thermal conductivity and specific heat capacity decrease dramatically as temperature deviates from the *T_m_* (307.75 K at 8 MPa). When *q_w_* continuously increases at a fixed mass flux, the deviation in *T_b_* from *T_in_* will increase, and the thermophysical properties of CO_2_ will deteriorate. As a result, the *h* of the CO_2_-cooled channel decreases rapidly with the increase in *q_w_*.

#### 3.2.2. Pressure Drop and Pumping Power

**Figure 13:**
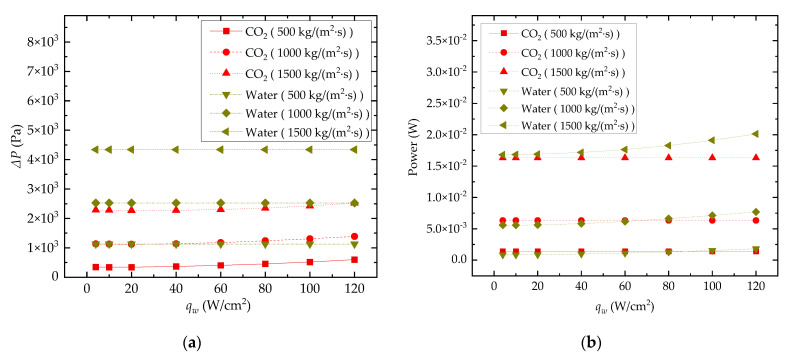
Effect of *q_w_* on ΔP and pumping power consumption: (**a**) pressure drop; (**b**) power.

Figure 13a,b illustrate the effect of *q_w_* on channel pressure drop (Δ*P*) and pump power consumption (*W*), respectively. As shown in Figure 13a, the ΔP of the water-cooled channel is significantly higher than that of the CO_2_-cooled channel. This result also shows that the variation in *q_w_* has little impact on the channel pressure drop in water-cooled cases. However, for the CO_2_-cooled channel, the Δ*P* curves show a slight upward trend as *q_w_* increases. This is caused by the increase in *T_b_* of CO_2_, which will lead to a decrease in the density and dynamic viscosity of CO_2_. The influence of these two parameters on the channel Δ*P* is opposite and the combined effect is that the Δ*P* of the CO_2_-cooled channel increases slightly with increasing *q_w_*. Although the Δ*P* in the water-cooled channel is higher, the difference in pump power consumption between the CO_2_-cooled and water-cooled channels is relatively small under the same mass flux, as shown in Figure 13b, because of the lower density of the CO_2_ compared to water.

#### 3.2.3. Entropy Generation Rate

**Figure 14:**
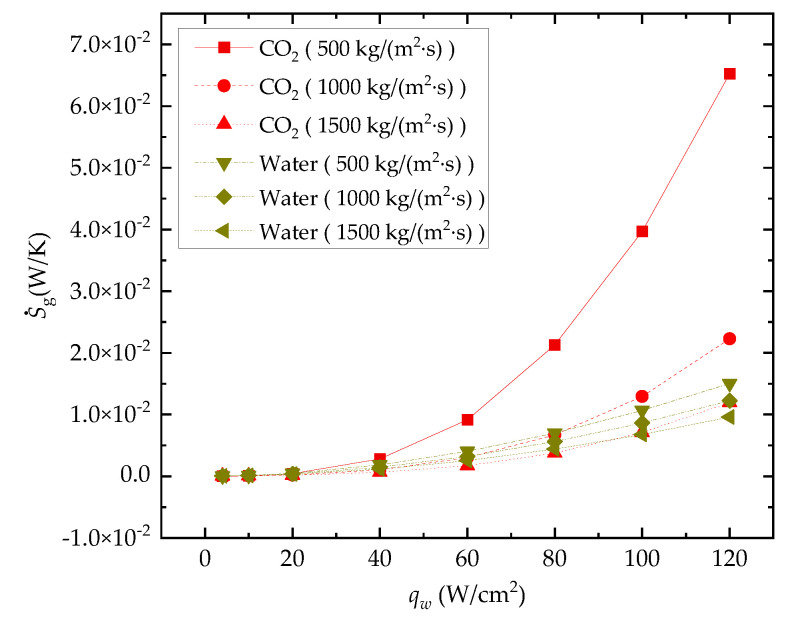
Effect of heat flux on ${\dot{S}}_{g}$.

The effect of *q_w_* on entropy generation rate ${\dot{S}}_{g}$ is presented in Figure 14. It can be seen from the comparison result that with the increase in heat flux density, the ${\dot{S}}_{g}$ of both the water-cooled channel and the CO_2_-cooled channel show an upward trend, but the increase rate of the CO_2_-cooled channel is higher. This means that the irreversible loss of the CO_2_-cooled channel is more affected by the heat flux. It can also be seen from the comparison of the curves that the accelerating upward trend of ${\dot{S}}_{g}$ with the increase in *q_w_* for the CO_2_-cooled microchannel is more significant. This is because an increase in *q_w_* leads to a greater deviation from the pseudocritical point of the CO_2_ fluid state, which will also weaken the heat transfer performance of the channel. 

### 3.3. Comparative Analysis of Zigzag Channels

Zigzag is a common channel type in industrial applications. The existence of bends along the channel can increase the intensity of the turbulence of the fluid and enhance the mixing of the wall fluid and the mainstream, to achieve the purpose of strengthening heat transfer. In this analysis, 90°, 120°, 150°, and 180° (straight channel) were used for the comparative study to investigate the heat transfer enhancement characteristic for zigzag channels cooled by water and CO_2_. In this comparative analysis, four inlet temperatures (*T_in_* = 308, 310, 315, and 320 K) and a fixed outlet pressure of *P_out_* = 8 MPa are considered for the CO_2_-cooled channel. For the water-cooled microchannel cases, fixed *T_in_* = 308 K and *P_out_* = 0.1 MPa are used, and *G* = 50~500 kg/(m^2^·s) and *q_w_* = 40,000 W/m^2^ are set for both water-cooled and CO_2_-cooled cases.

#### 3.3.1. Comparative Study of h under Different Angles of Bend in The Zigzag Channel

**Figure 15:**
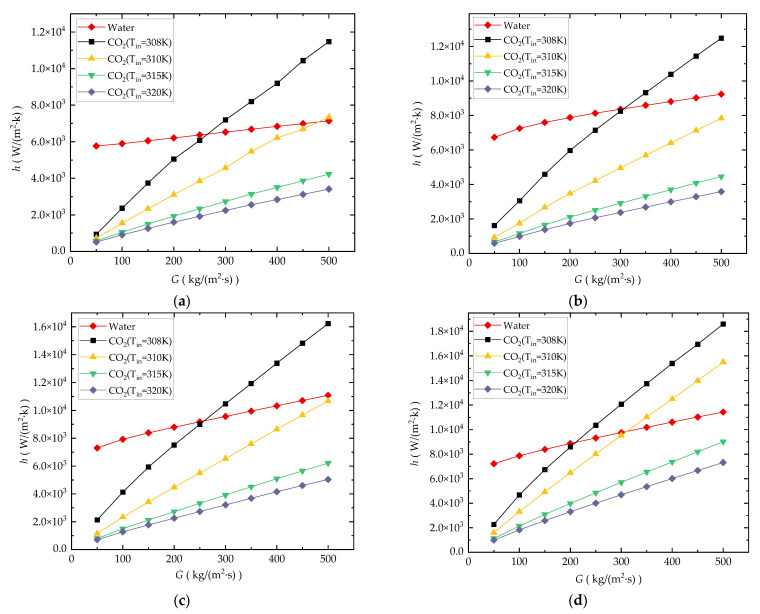
Comparison of *h*: (**a**) straight channel; (**b**) *θ* = 150°; (**c**) *θ* = 120°; (**d**) *θ* = 90°.

Figure 15a–d show the comparison of the *h* in CO_2_-cooled and water-cooled channels with four different bending angles, including the straight channel (*θ* = 180°). The *h* of the water-cooled channel for all four bending angle channel types is greater compared to the CO_2_-cooled channel under low mass flux. This means that the heat transfer performance of CO_2_ is worse than that of water at a relatively low mass flux. This is because the lower mass flux will aggravate the deviation in the *T_b_* and *T_w_* from the *T_in_* of the CO_2_ fluid, resulting in a greater deviation in the state of CO_2_ from the pseudocritical point. The thermophysical parameters (specific heat and thermal conductivity) of CO_2_ are greatly reduced. 

However, the increased rate of *h* with *G* of the CO_2_-cooled channel is higher than that of the water-cooled channel for all four bending angle channel types. This result means that the increase in mass flux has a greater impact on *h* of the CO_2_-cooled channel than on the water-cooled one. This is because increasing the mass flux not only increases the *Re* of the microchannel and enhances turbulence, but also makes the *T_b_* of CO_2_ closer to *T_m_*, and the heat transfer performance can be further improved.

**Figure 16:**
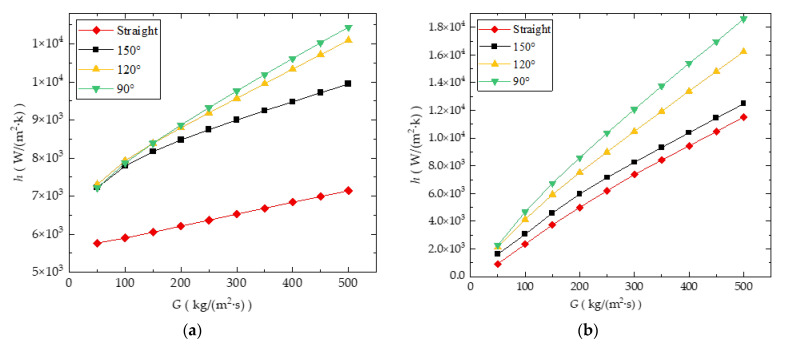
Comparison of the effect of bend angle on *h*: (**a**) water-cooled channel; (**b**) CO_2_-cooled channel (*T_in_* = 308 K).

Figure 16a,b provide the comparison of *h* in different bend angle cases for CO_2_-cooled and water-cooled microchannels of zigzag type, respectively. Obviously, the comparison results in these figures indicate that for both CO_2_-cooled and water-cooled microchannels, reducing the turning angle is conducive to enhancing heat transfer. However, the comparison result also shows that the existence of bends has a more significant impact on the water-cooled channel. Compared to the straight channel case, the *h* of the zigzag channel with *θ* = 150° increases significantly. However, the improvement in heat transfer performance of water-cooled cases by further reducing *θ* is no longer as significant as the transition from straight to zigzag type. 

**Figure 17:**
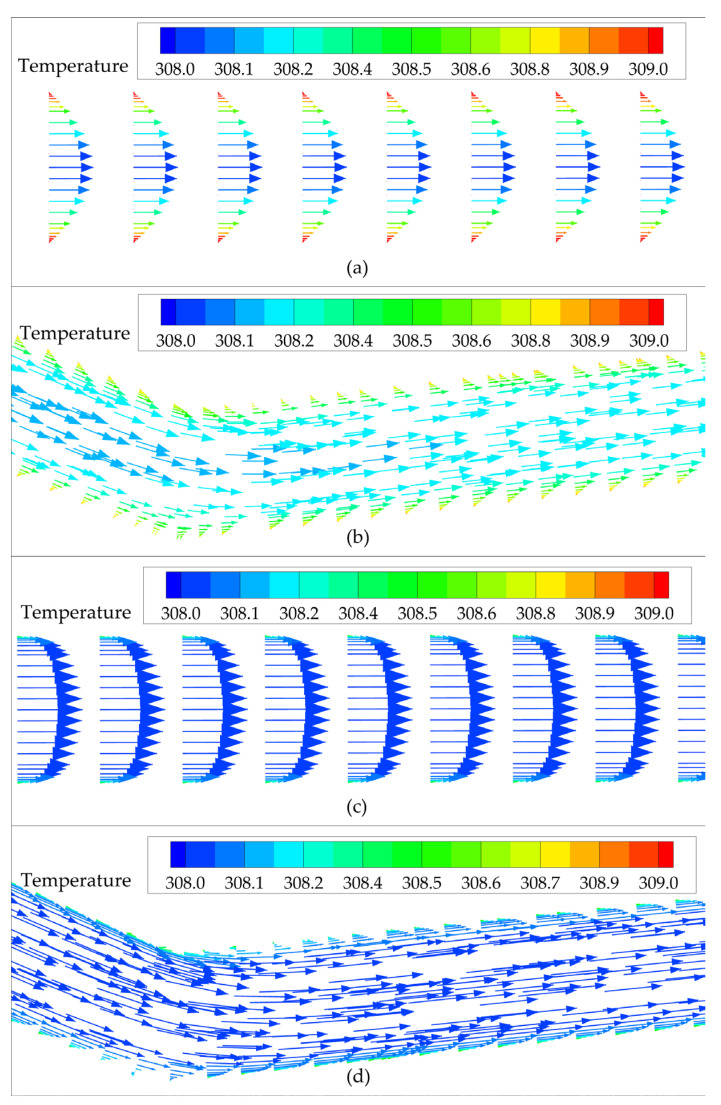
Velocity vector diagram of the cross section of microchannel (*G* = 500 kg/(m^2^·s), *T_in_* = 308 K): (**a**) water-cooled straight channel; (**b**) water-cooled zigzag channel (*θ* = 150°); (**c**) CO_2_-cooled straight channel; (**d**) CO_2_-cooled zigzag channel (*θ* = 150°).

In the analysis cases, the fluid of the water-cooled channel is in a laminar state with low *Re* (6.17~61.7), and the existence of bends in the zigzag channel can greatly improve the intensity of turbulence and significantly thin the thermal boundary layer compared to the straight channel, as illustrated in Figure 17a,b. For the CO_2_-cooled channel, the heat transfer enhancement effect produced by using a zigzag channel instead of the straight channel is not as great as that of the water-cooled channel. This is because the sCO_2_-cooled straight channel has a thinner thermal boundary layer than the water-cooled straight channel, as illustrated in Figure 17c,d, and the variation in the thickness of the thermal boundary layer caused by the bending in the channel is not as significant as that of the water-cooled channel. 

#### 3.3.2. Comparative Study of ${\dot{S}}_{g}$ under Different Angles of Bend in the Zigzag Channel

**Figure 18:**
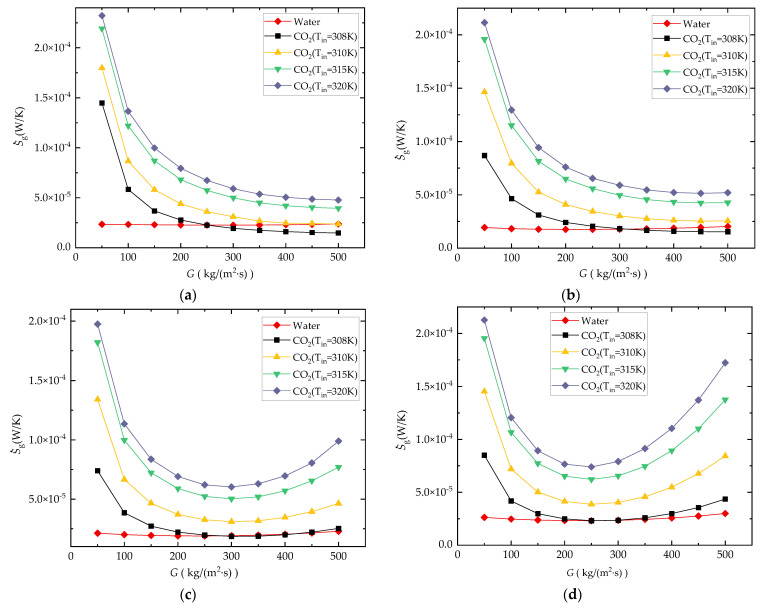
Comparison of ${\dot{S}}_{g}$: (**a**) straight channel; (**b**) *θ* = 150°; (**c**) *θ* = 120°; (**d**) *θ* = 90°.

The existence of and reduction in the bend angle of the zigzag channel can enhance thermal performance, but cause an increase in the channel pressure drop, which leads to an increase in pump power consumption. This means that the thermal entropy generation decreases while the flow entropy generation increases. The total entropy generation rate ${\dot{S}}_{g}$ is illustrated in Figure 18a–d for the water-cooled and sCO_2_-cooled microchannel in different *θ* cases.

For the CO_2_-cooled microchannel of straight type and zigzag type with *θ* =150°, as shown in Figure 18a,b, the ${\dot{S}}_{g}$ shows a decreased trend as *G* increases from 50 to 500 kg/(m^2^·s), which means that the decreasing amplitude of thermal entropy generation caused by the increasing flow flux is greater than the increasing amplitude of flow entropy generation. For the CO_2_-cooled microchannel of zigzag type with *θ* =120° and *θ* =90°, as shown in Figure 18c,d, the ${\dot{S}}_{g}$ curve presents a U shape. The minimum value of ${\dot{S}}_{g}$ occurs when *G* is between 250 and 300 kg/(m^2^·s). With the decrease in *θ* of the zigzag microchannel, the proportion of the flow entropy generation in total entropy generation becomes larger. Figure 18 also shows that when *T_in_* = 310, 315, and 320 K, ${\dot{S}}_{g}$ of the CO_2_-cooled case is larger than that of the water-cooled case in the whole analysis flow flux range for all straight and zigzag microchannels. In this case, there is no advantage in using CO_2_ to replace water as a coolant. When *T_in_* = 308 for straight type and zigzag type with *θ* =150°, the ${\dot{S}}_{g}$ of the CO_2_-cooled microchannel is lower than that of the water-cooled channel under high-flow flux conditions.

**Figure 19:**
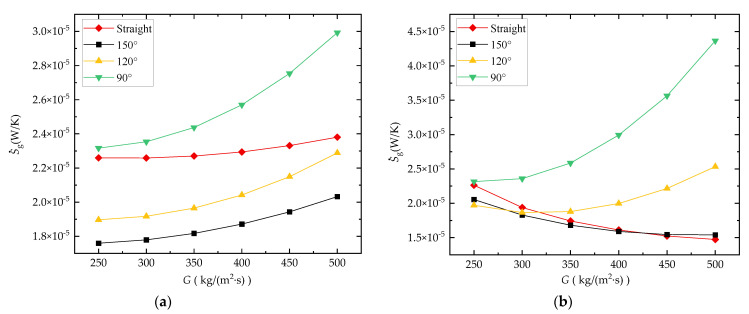
Comparison of the effect of bend angle on ${\dot{S}}_{g}$: (**a**) channel cooled by water; (**b**) channel cooled by CO_2_ (*T_in_* = 308 K at *P_out_* = 8 MPa).

Figure 19a,b show the effect of *θ* on ${\dot{S}}_{g}$ of the water-cooled and sCO_2_-cooled microchannel with *T_in_* = 308 K, respectively. It can be seen from the analysis result that in water-cooled cases, the zigzag channel with *θ* = 150° has the best comprehensive performance (lowest valve of ${\dot{S}}_{g}$), followed by *θ* = 120° and straight channels, and the zigzag channel with *θ* = 90° is the worst. For the cases of sCO_2_-cooled channels, the ${\dot{S}}_{g}$ of the straight channel and zigzag channel with *θ* =150° is better, and with a reduction in the value of *θ*, the ${\dot{S}}_{g}$ of the zigzag channel gradually increases. This analysis result also shows that although the existence of bends in the microchannel can improve the *h* of the sCO_2_-cooled microchannel, it is not beneficial to its comprehensive performance evaluated by ${\dot{S}}_{g}$. However, the existence of obtuse bends in the channel can improve the comprehensive performance to some extent for the water-cooled microchannel.

## 4. Conclusions

A numerical comparative study was carried out for sCO_2_-cooled and water-cooled straight and zigzag (*θ* = 90°, 120°, and 150°) microchannels. The mass flux (*G*) ranges from 50 to 1000 kg/(m^2^·s) and the heat flux (*q_w_*) ranges from 40,000 to 120,000 W/m^2^ for both coolant-based cooling cases. The inlet temperature (*T_in_*) for CO_2_-cooled microchannels ranges from 306 K to 320 K with the operating pressure 8 MPa, which covers the pseudocritical point. The inlet temperature of the water-cooled microchannel is 308 K with the operating pressure 0.1 MPa. The following conclusions were obtained:

(1) Using sCO_2_ has advantages over water in thermal and hydraulic performance in microchannels due to its excellent thermophysical properties nearby the pseudocritical point. Taking sCO_2_ as a coolant makes it possible to reduce the average temperature of the heating surface (*T_hsur_*) to 0.5~0.7 K and enhance the heat transfer performance in contrast to water. The entropy generation rate (${\dot{S}}_{g}$) of the straight microchannel cooled with sCO_2_ can reach 0.58~0.69 times the one cooled with water. 

(2) Using CO_2_ replacing water as the coolant can improve microchannel thermal and hydraulic performance, but the premise lies in adopting a reasonable inlet temperature, working pressure, and adequate channel mass flux according to the heat load to keep the CO_2_ state near the pseudocritical point. 

(3) In comparison with straight channels, zigzag channels can enhance heat transfer, but this will also increase the channel flow resistance. As for the water-cooled case, the zigzag channel with θ = 150° had the best comprehensive performance represented by ${\dot{S}}_{g}$, while for the sCO_2_-cooled case, the straight channel had the best comprehensive performance.

## Figures and Tables

**Table 1:** Boundary conditions for both coolants.

Coolants	*G* (kg/(m^2^·s))	*P_out_* (MPa)	*Q_w_*(W/m^2^)	*T_in_* (K)
sCO_2_	50~1000	8	40,000~120,000	306, 307, 308, 310, 315, 320
water	50~1000	0.1	40,000~120,000	308

## Data Availability

Not applicable.

## Nomenclature

*A*	area (m^2^)	Greek symbols	
*C*	wet circumference (m)	ρ	density (kg/m^3^)
$d_{h}$	hydraulic diameter (m)	μ	dynamic viscosity (Pa·s)
$\vec{g}$	gravity vector (m/s^2^)	*θ*	bend angle
*G*	mass flux (kg/(m^2^·s))		
*h*	average heat convection coefficient (W/(m^2^·K))	Subscripts	
*k*	thermal conductivity (W/(m·K))	*b*	bulk
$\dot{m}$	mass flow rate (kg/s)	*eff*	effective
*Nu*	Nusselt number	*hsur*	heated surface
*P*	pressure (Pa)	*in*	inlet
ΔP	pressure difference (Pa)	*m*	pseudocritical point
*q*	heat flux (W/m^2^)	*out*	outlet
*Q*	heat (W)	*t*	turbulent
*Re*	Reynolds number	*w*	wall
${\dot{S}}_{g}$	entropy generation rate (W/K)		
*T*	temperature (K)		
$\vec{v}$	velocity vector (m/s)		
